# A comparative study of osteopontin and MMP-2 protein expression in peripheral and central giant cell granuloma of the jaw^☆^

**DOI:** 10.1016/j.bjorl.2017.11.006

**Published:** 2017-12-27

**Authors:** Nooshin Mohtasham, Nasrollah Saghravanian, Bahareh Fatemi, Mehdi Vahedi, Monavar Afzal-Aghaee, Hamideh Kadeh

**Affiliations:** a Oral and Maxillofacial Disease Research Center, Mashhad University of Medical Sciences, School of Dentistry, Department of Oral and Maxillofacial Pathology, Mashhad, Iran; b Kerman University of Medical Sciences, School of Dentistry, Department of Endodontics, Kerman, Iran; c Kerman University of Medical Sciences, School of Dentistry, Department of Periodontology, Kerman, Iran; d Mashhad University of Medical Sciences, Social Medicine Specialist, Mashhad, Iran; e Oral and Dental Disease Research Center, Zahedan University of Medical Science, School of Dentistry, Department of Oral & Maxillofacial Pathology, Zahedan, Iran

**Keywords:** Osteopontin, MMP-2, PGCG, CGCG, Immunohistochemistry, Osteopontina, MMP-2, PGCG, CGCG, Imuno-histoquímica

## Abstract

**Introduction:**

Oral peripheral and central giant cell granulomas are lesions with little-known etiology and pathogenesis.

**Objective:**

The aim of this study was to compare matrix metalloproteinases-2 and osteopontin protein expression in the multinucleated giant cells and mononuclear cells of the peripheral and central giant cell granuloma lesions.

**Methods:**

In this retrospective study, the presence of matrix metalloproteinases-2 and osteopontin in 37 cases of central giant cell granuloma and 37 cases of peripheral giant cell granuloma paraffin blocks were assessed by streptavidin-biotin immunohistochemistry. Independent sample *t*-test, Chi-square, Mann–Whitney tests and Spearman's rank correlation coefficient were used.

**Results:**

The osteopontin was expressed in both multinucleated giant cells and mononuclear cells in all cases of peripheral and central giant cells granulomas. However, the matrix metalloproteinases-2 expression was positive in 86.5% of giant cells and it was positive in all of mononuclear cells in peripheral giant cells granuloma. In central giant cells granulomas, 91.8% of giant cells and all mononuclear cells were positive for matrix metalloproteinases-2 marker. Percentage and Intensity of staining were significantly higher in central than peripheral giant cells lesions, for both markers (*p* ˂ 0.05).

**Conclusion:**

This study showed that the expression of osteopontin in giant cells supports the theory of osteolcastic nature of these cells. Also, the presence of osteopontin and matrix metalloproteinases-2 in mononuclear cells may indicate the monocyte-macrophage origin of these cells, as the differentiation of the precursors of the mononuclear stromal monocyte/macrophage to osteoclasts is possibly affected by the expression of osteolytic factors. Also, may be differences in biological behaviors of these lesions are associated with the level of osteopontin and matrix metalloproteinases-2 expression.

## Resumo

**Introdução:**

Os granulomas periféricos e centrais de células gigantes são lesões com etiologia e patogênese pouco conhecidas.

**Objetivo:**

Comparar a expressão das proteínas metaloproteinases da matriz-2 e osteopontina nas células gigantes multinucleadas e células mononucleares no granuloma periférico e central de células gigantes.

**Método:**

Neste estudo retrospectivo, a presença de metaloproteinases da matriz-2 e osteopontina em 37 casos de granuloma central de células gigantes e 37 casos de granuloma periférico de células gigantes em blocos de parafina foi avaliada por imuno-histoquímica pela estreptavidina-biotina. Foram usados teste *t* para amostra independente, teste de qui-quadrado, Mann-Whitney e coeficiente de correlação de Spearman.

**Resultados:**

A osteopontina foi expressa em células gigantes multinucleadas e células mononucleares em todos os casos de granuloma periférico de células gigantes e granuloma central de células gigantes. No entanto, a expressão de metaloproteinases da matriz-2 foi positiva em 86,5% de células gigantes e foi positiva em todas as células mononucleares em granuloma periférico de células gigantes. Em granuloma central de células gigantes, 91,8% das células gigantes e todas as células mononucleares foram positivas para o marcador metaloproteinases da matriz-2. A porcentagem e intensidade de coloração em granuloma central de células gigantes foram significantemente maiores do que em granuloma periférico de células gigantes para ambos os marcadores (*p* ˂ 0,05).

**Conclusão:**

Este estudo mostrou que a expressão de osteopontina em células gigantes apoia a teoria da natureza osteoclástica dessas células. Além disso, a presença de osteopontina e metaloproteinases da matriz-2 em células mononucleares pode indicar a origem dos monócitos-macrófagos dessas células, uma vez que a diferenciação dos precursores do monócito/macrófago estromal mononuclear em osteoclastos é possivelmente afetada pela expressão de fatores osteolíticos. Além disso, as diferenças nos comportamentos biológicos dessas lesões estão associadas ao nível de expressão de osteopontina e metaloproteinases da matriz-2.

## Introduction

Peripheral giant cell granuloma (PGCG) is a relatively common lesion observed as a red or purple nodular mass on the gingiva or edentulous alveolar ridge.^1^, ^2^, ^3^ This lesion originates from the periodontal ligament and grows slowly.^4^, ^5^, ^6^ PGCG can occur at any age, especially in the sixth and fifth decades of life, with little tendency for females.^1^

Central giant cell granuloma (CGCG) is less common than PGCG and occurs centrally in the jaw bone.^7^ Radiographically, this lesion is observed as unilocular or multilocular radiolucency with specified limits.^1^, ^7^ It has different clinical features and may be a slow-growing asymptomatic lesion or a painful lesion with rapid growth and high recurrence.^8^, ^9^

Both PGCG and CGCG have similar histopathologic features and are characterized by the presence of Multinucleated Giant Cells (MGCs) in a background of mononuclear mesenchymal cells. However, despite these similarities, the two lesions are different in terms of biological behavior.^7^, ^8^, ^9^, ^10^ CGCG is a more aggressive lesion with a tendency to rapid growth, high recurrence, root resorption and bone perforation; while PGCG is a lesion with low recurrence, and in some cases may cause bone surface resorption.^7^, ^9^ Despite the various studies in this regard, the reason for different clinical behaviors of these lesions is unknown.^1^

On the other hand, although multi-nuclear giant cells are the hallmark of these lesions, the histogenesis of the giant cells has not been specified yet.^5^, ^7^, ^11^ Some investigators believe that the giant cells show the immunohistochemistry characteristics of osteoclasts,^5^, ^7^ while others have suggested the phagocytic and endothelial cells origin for these cells.^8^, ^12^ It is also indicated that the stromal mononuclear cells play an important role in the evolution of giant cells.^13^, ^14^

Osteopontin is a non-collagenous protein and a highly phosphorylated sialoprotein with high capacity to bind to calcium, and is produced by differentiating osteoblasts, differentiated osteoblasts, osteocytes, and osteoclasts.^15^ Osteopontin plays an important role in physiological bone remodeling, especially bone resorption through modulating.^16^ It can also play an important role in the formation of chronic inflammation, granuloma formation, migration of histocytes, and phagocytosis.^17^

Matrix metalloproteinases (MMP) are a family of zinc-dependent endopeptidases which are able to degrade organic matrix in physiological PH. Previous studies have suggested that MMPs are involved in bone resorption process, and MMP2 and MMP9 (A and B gelatinases) can be produced by osteoblasts or osteoclasts.^18^ It is reported that MMP-9 plays an important role in the processes of angiogenesis, bone resorption and regulating non-mineralized bone matrix proteolysis.^19^

In giant cell lesions, not only the giant cells but also stromal cells are involved in the production of tissue-destructive enzymes such as MMPs. However, few studies have been conducted on the role of MMPs in the pathogenesis of Giant cell lesions.^19^, ^20^

According to our knowledge, no similar study has been done on comparing the MMP-2 and osteopontin expression in the peripheral and central giant cell granuloma lesions in the jaws. Considering the microscopic similarities of PGCG and CGCG and differences in their biologic behavior, we evaluated the expression of MMP-2 and osteopontin proteins in these two lesions by immunohistochemistry in this study, to possibly confirm the osteoclastic phenotype of MGCs and the relationship of this immunohistochemical divergence with different behaviors of PGCG and CGCG.

## Methods

In this retrospective study following approval of the local Ethics Committee (910127) a total of 74 cases included 37 PGCG and 37 cases of CGCG were evaluated immunohistochemically for osteopontin and MMP-2 protein expression.

A streptavidin-biotin immunohistochemistry standard method was used. For immunohistochemical staining, 4 μm sections were cut from paraffin blocks; sections deparaffinized in Xylene and rehydrated using a graded ethanol. The tissue was incubated in 30% hydrogen peroxide-methanol for 30 min to block endogenous peroxidase activity and the slides were washed in Phosphate Buffered Saline (PBS). For antigen retrieval, the slides were immersed in citrate solution and were microwaved for 15 min. Following the sections were incubated with protein block in order to eliminate background staining. Then the slides were incubated with primary antibodies of MMP-2 (Code: NCL-MMP2-507, Clone: 17B11, Novocastra, United Kingdom; Dilute 1:50) and osteopontin (Code: NCL-O-PONTIN, Clone: OP3N, Novocastra, United Kingdom; Dilute 1:80) according to manufacturer's instruction. The sections were washed 3 times with PBS at room temperature. Immune complexes were treated with secondary antibody and detected by streptavidin peroxidase (Novolink Polymer Detection System: Code RE7230-K). Immunoreactivity was visualized with diaminobenzidine and was counterstained with Mayer hematoxylin and after drying, the sections were mounted. Sections of ulcerative colitis and gallbladder were used as positive control for MMP-2 and osteopontin respectively and as a negative control primary antibody was omitted.

For assessment of MMP-2 and osteopontin positivity, the number of positive cells was counted in 5 microscopic fields in hot spot (the most populated areas by cells) with a magnification of 100 with light microscope (Leica Galen III, USA). Percentage of cell staining was scored according to others studies^21^, ^22^: negative (no staining), 0%–5% stained cells (−), 5%–25% stained cells (+), 25%–50% stained cells (++), 50%–75% stained cells (+++), 75%–100% stained cells (++++). Intensity of staining was scored as: negative (no staining), mild (light brown staining of the cells), severe (dark brown staining of the cells) and moderate (between mild and severe staining of the cells).

### Statistical analysis

Data analysis was performed in SPSS version 21 (SPSS Inc, Chicago, IL) using Kolmogorov–Smirnov test to evaluate normal distribution of quantitative data, independent sample *t*-test to compare normally distributed quantitative variables (age between PGCG group and CGCG group), Chi-square test to qualitative variables (gender between PGCG group and CGCG group) and Mann–Whitney test for other qualitative variables (cell staining percentage and staining intensity in PGCG and CGCG). *p*-value less than 0.05 was considered statistically significant.

## Results

In this study, 37 PGCG and 37 CGCG cases were examined. Demographic data of the subjects are separately shown in Table 1. The mean age of PGCG cases was 35.08 ± 20.63, and in CGCG it was 26.9 ± 15.14, but this difference was not statistically significant (*p* = 0.06; Independent sample *t*-test). Also, about 60% of cases in both PGCG and CGCG occurred in females, but the difference between the two groups was not statistically significant (*p* = 0.47; Chi-Square Test).

**Table 1:** Summary of the demographic data of PGCG and CGCG lesions.

Lesions	Age			Gender		
	Mean ± SD	Range	*p*-value	Male	Female	*p*-value
PGCG	35.08 ± 20.63	10–71	0.06	15 (40)	22 (60)	0.47
CGCG	26.9 ± 15.14	6–76		13 (38.24)	21 (61.76)	

PGCG, peripheral giant cell granuloma; CGCG, central giant cell granuloma.

**Figure 1:**
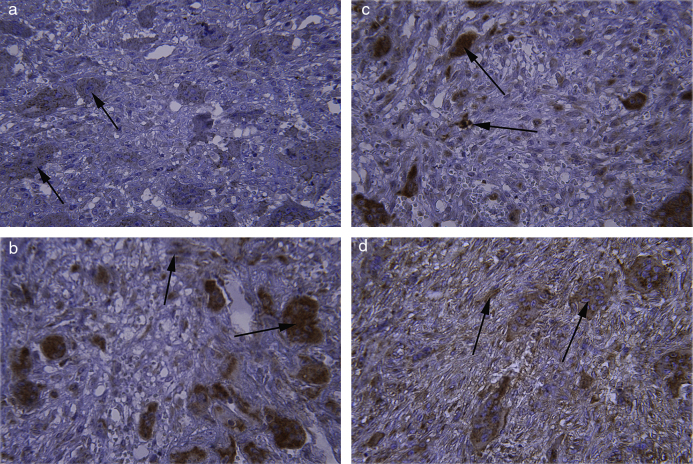
Immunohistochemical staining of (a) MMP-2 in giant cells of CGCG with mild intensity staining (×400). (b, c) Osteopontin in giant cells and mononuclear cells of PGCG with severe intensity staining (×400). (d) Osteopontin in giant cells and mononuclear cells of PGCG with moderate intensity staining (×400).

High percentage of giant cells and mononuclear cells in both lesions were positive for MMP-2 (Table 2) (Fig. 1a). The MMP-2 staining was not significantly different between the mononuclear and giant cells in CGCG (*p* = 0.16), but The MMP-2 staining was significantly different between the mononuclear and giant cells in PGCG (*p* ˂ 0.000).

**Table 2:** Immune reactivity of the MMP-2 in lesions of PGCG and CGCG.

	PGCG		CGCG	
	MGC *n* (%)	MC *n* (%)	MGC *n* (%)	MC *n* (%)
Negative	5 (13.5)	0	3 (8.1)	0
+	22 (59.5)	9 (24.3)	7 (18.9)	6 (16.2)
++	8 (21.6)	22 (59.5)	14 (37.8)	13 (35.6)
+++	1 (2.7)	5 (13.5)	12 (32.4)	17 (45.9)
++++	1 (2.7)	1 (2.7)	1 (2.7)	1 (2.7)

*p*-value	0.000		0.16	

PGCG, peripheral giant cell granuloma; CGCG, central giant cell granuloma; MGC, multinucleated giant cell; MC, mononuclear cell.

According to the Mann–Whitney test, the Median of MMP-2 staining in the giant cell was statistically significant between two lesions (*p* ˂ 0.000). This difference was also statistically significant in mononuclear cells between the CGCG and PGCG (*p* = 0.015).

All giant and mononuclear cells were positive for osteopontin in PGCG and CGCG (Table 3) (Fig. 1b–d). The percentage of cell staining for osteopontin was higher in giant cells than the mononuclear cells in both lesions, but this difference was not statistically different in both groups (PGCG – *p* = 0.56; CGCG – *p* = 0.18).

**Table 3:** Immunoreactivity of the osteopontin in lesions of PGCG and CGCG.

	PGCG		CGCG	
	MGC *n* (%)	MC *n* (%)	MGC *n* (%)	MC *n* (%)
Negative	0	0	0	0
+	5 (13.5)	5 (13.5)	0	1 (2.7)
++	12 (32.4)	16 (43.2)	1 (2.7)	5 (13.5)
+++	11 (29.7)	8 (21.6)	13 (35.1)	12 (32.4)
++++	9 (24.3)	8 (21.6)	23 (62.2)	19 (51.4)

*p*-value	0.56		0.18	

PGCG, peripheral giant cell granuloma; CGCG, central giant cell granuloma; MGC, multinucleated giant cell; MC, mononuclear cell.

According to the Mann–Whitney test, the Median of osteopontin staining in giant cells was statistically different between two lesions (*p* ˂ 0.000). Also in mononuclear cells, the difference was statistically different between PGCG and CGCG (*p* ˂ 0.000).

Table 4 shows the staining intensity of giant cells and mononuclear cells for osteopontin in the lesions. According to this table, the difference of osteopontin staining intensity between the mononuclear and multinuclear giant cells in CGCG (*p* = 0.65) and between the mononuclear and giant cells in PGCG (*p* = 0.82) was not statistically significant.

**Table 4:** Staining intensity of the osteopontin in lesions of PGCG and CGCG.

OPN	PGCG		CGCG	
	MGC *n* (%)	MC *n* (%)	MGC *n* (%)	MC *n* (%)
Negative	0	0	0	0
Mild	5 (13.5)	4 (10.8)	1 (2.7)	3 (8.1)
Moderate	25 (67.6)	26 (70.3)	18 (48.6)	17 (45.9)
Severe	7 (18.9)	7 (18.9)	18 (48.6)	17 (45.9)

*p*-value	0.82		0.65	

PGCG, peripheral giant cell granuloma; CGCG, central giant cell granuloma; MGC, multinucleated giant cell; MC, mononuclear cell.

However, according to Mann–Whitney test, median staining intensity of osteopontin in giant cells between the two lesions was statistically significant (*p* = 0.003). Also, the difference was statistically significant in mononuclear cells between the two lesions (*p* = 0.035).

Table 5 shows the staining intensity of giant cells and mononuclear cells for MMP-2 in the lesions. MMP-2 staining intensity between multinuclear and mononuclear giant cells in CGCG (*p* = 0.39) and between multinuclear and mononuclear giant cells in PGCG (*p* = 0.34) was not significant.

**Table 5:** Staining intensity of the MMP-2 in lesions of PGCG and CGCG.

MMP-2	PGCG		CGCG	
	MGC *n* (%)	MC *n* (%)	MGC *n* (%)	MC *n* (%)
Negative	3 (8.1)	0	3 (8.1)	0
Mild	23 (62.2)	24 (64.9)	11 (29.7)	12 (32.4)
Moderate	11 (29.7)	13 (35.1)	23 (62.2)	24 (64.9)
Severe	0	0	0	1 (2.7)

*p*-value	0.93		0.39	

PGCG, peripheral giant cell granuloma; CGCG, central giant cell granuloma; MGC, multinucleated giant cell; MC, mononuclear cell.

The median staining intensity of MMP-2 in multinuclear giant cells was not statistically significant between the two lesions (*p* = 0.14). But the difference was not statistically significant in mononuclear cells between the two lesions (*p* = 0.05; Mann–Whitney)

Also Spearman's rank correlation coefficient showed a significant correlation between osteopontin and MMP-2 in both PGCG (*p* = 0.004, *r* = 0.332) and CGCG (*p* = 0.019, *r* = 0.273).

## Discussion

Peripheral and central giant cell granulomas include non-neoplastic lesions with little-known etiology and pathogenesis. Various histological, immunohistochemical, enzymatic and ultrastructural studies have been conducted to determine the role and origin of giant cells in these lesions, but their nature still remains unknown.^7^ Thus, we decided to compare the two proteins involved in bone and connective tissue resorption (osteopontin and MMP-2) in giant cells and mononuclear cells of these lesions. In this study, all giant cells and mononuclear cells of CGCG PGCG were positive for osteopontin. Also for the MMP-2, most multinucleated giant and mononuclear cells in both lesions were positive for this marker, and this was significantly higher in CGCG compared to PGCG in both multinuclear and mononuclear cells.

In 2011, Matos et al.^19^ examined the expression of MMP-9 in the central and peripheral giant cell granuloma lesions in jaws. The results revealed higher MMP-9 expression in central giant cell granuloma and suggested that MMP-9 may play an important role in the process of osteoclastogenesis of the CGCG lesions. Also, in the present study, given the higher presence of MMP-2 in CGCG lesions, this marker may be involved in the osteoclastogenesis process of CGCG lesions.

In 2010, Tobon et al.^20^ examined the relationship between the expression of MMP-9 and MMP-1 with the clinical behavior of giant cell lesions in invasive and non-invasive forms. The results showed that both proteases are significantly higher in invasive lesions, which is in compliance with our study in terms of the relative differences in the biologic behavior of peripheral and central giant cell granuloma lesions, as MMP-2 was higher in invasive lesions in our study.

In a study conducted in 2010 by Friedrich et al.,^23^ the factors indicating the differentiation and activity of osteoclasts, such as MMP-9, were evaluated using the Microarray technique. These factors were found in all studied lesions including giant cell lesions of the jaw, tendon sheaths, and salivary glands. The results of this study showed that cellular ingredients for all lesions is independent of the location and giant cell lesions at all site include similar osteolytic proteases and express metabolic cytokines that affect the bone metabolism. Despite differences in method, expression of MMP-9 as an osteolytic factor in giant cell lesions of jaw is consistent with our study.

In a study by Liu et al.^24^ to evaluate the characteristics of osteoclastic giant cell in giant cell lesions of the jaw, Immunohistochemical (IHC) studies showed that giant cells and a number of mononuclear cells in these lesions were largely positive for MMP-9. Also, in their study, MGCs in giant cell lesions of the jaw showed osteoclastic characteristics. Also, in our study, the expression of MMP-2 in multinucleated giant cells may indicate the role of giant cells in osteolytic and proteolytic activities in these lesions.

In a study conducted by Carlson et al.,^17^ 22 cases of granulomatous lesions were examined in terms of the expression levels of osteopontin, and the results showed an over expression of osteopontin mRNA in multinuclear giant cells. In our study, the percentage of staining of osteopontin in multinucleated giant cells was higher compared to mononuclear cells in peripheral and central giant cell granulomas, but the difference was not significantly different. The difference in results may be due to different techniques of these two studies.

In 2011, Torabinia et al.^7^ reported the expression of TRAP (indicated the osteoclastic activity of the cells) in multinucleated giant cells and a number of mononuclear cells in peripheral and central giant cell granuloma lesions and suggested that giant cells represent osteoclastic phenotype in PGCG and CGCG. Also, a group of stromal mononuclear cells that showed this marker can be considered as progenitors of giant cells. In our study, osteopontin, which is an osteoclastic marker, was expressed higher in multinuclear cells than mononuclear cells.

In a study by Rabinovich et al.,^25^ the expression of MMP2, 9 in stromal cells of giant cell tumor in bone indicates the role of these cells in stromal gelatin degradation and bone invasion. Similarly, in our study, stromal cells and giant cells in the central giant cell granuloma expressed MMP-2, which indicates the role of mononuclear cells and giant cells in destroying the bone matrix.

Also, in a study on examining the osteoclastic markers of RANKL and osteoprotegrin in PGCG, Fanourakis et al.^6^ indicated the osteoclastic nature of giant cells, but the possible osteoclastic nature of the stromal monocytes was ambiguously reported. This study is similar to the current study regarding indicating the osteoclastic nature of giant cells.

Contrary to the current study which indicates that the osteopontin and MMP-2 markers could explain the different clinical behaviors of CGCG and PGCG, in a study by Souza et al.,^26^ the expression of P53, MDM-2, PCNA, Ki-67 in CGCG and PGCG was not significantly different and did not explain their different behavior. Also, in a study by Moradzadeh et al.^5^ in 2013 to examine the expression of Src protein in the central and peripheral giant cell granuloma, it was concluded that MGCs showed similarities with osteoclast in these lesions, and this marker may be used as a new therapeutic target for inhibiting the activity of osteoclasts in these legions. This study was similar to our study in terms of presence of an osteoclastic nature in giant cells in the mentioned lesions. But they suggest that Src marker does not explain the different biological behavior of PGCG and CGCG. In our study, both osteopontin and MMP-2 markers in CGCG were higher than PGCG and this can be indicative of the fact that differences in biological behaviors of these lesions are associated with the expression of osteolytic and proteolytic markers.

## Conclusion

According to the results of current study, the expression of osteopontin in giant cells supports the theory of osteolcastic nature of these cells. Also, the presence of this marker and MMP-2 in mononuclear cells may indicate the monocyte-macrophage origin of these cells, as differentiation of the precursors of the mono-nuclear stromal monocyte/macrophage to osteoclasts are possibly affected by the expression of osteolytic factors. Also, due to the significant difference between marker expression of osteopontin and MMP-2 in PGCG and CGCG, it can be said that differences in biological behaviors of these lesions are associated with the level of expression of osteolytic and proteolytic markers.

## Funding

This work was supported by the Research Council of Mashhad University of Medical Sciences (grant number: 910127).

## Conflicts of interest

The authors declare no conflicts of interest.
